# Correction: Chang et al. Exercise Guidelines in Pancreatic Cancer Based on the Dietz Model. *Cancers* 2025, *17*, 630

**DOI:** 10.3390/cancers18050789

**Published:** 2026-02-28

**Authors:** Philip J. Chang, Andrew E. Hendifar, Gillian Gresham, An Ngo-Huang, Paul E. Oberstein, Nathan Parker, Andrew L. Coveler

**Affiliations:** 1 Cedars-Sinai Medical Center, Los Angeles, CA 90211, USA; andrew.hendifar@cshs.org (A.E.H.); gillian.gresham@cshs.org (G.G.); 2 MD Anderson Cancer Center, Houston, TX 77030, USA; ango2@mdanderson.org; 3 New York University, New York, NY 10012, USA; paul.oberstein@nyulangone.org; 4 Moffitt Cancer Center, Tampa, FL 33612, USA; nathan.parker@moffitt.org; 5 University of Washington, Seattle, WA 98105, USA; acoveler@uw.edu

In the original publication [1], the Precision Promise Consortium was not included in the authorship. The authors apologize for any inconvenience caused and state that the scientific conclusions are unaffected. This correction was approved by the Academic Editor. The original publication has also been updated.
